# Postmenopausal Pregnancy Via Oocyte Donor and Suspected Focal Placenta Accreta: A Case Report

**DOI:** 10.7759/cureus.62332

**Published:** 2024-06-13

**Authors:** Rima Hajjar, Saladin A Cooper, Nila Elumalai, Hussain Hussain, Rebecca Patrizio, Abdulrahman Sinno, Anis Ahmad, Staci Marbin, Roberto Ruiz-Cordero, Michael J Paidas, Arumugam R Jayakumar

**Affiliations:** 1 Department of Obstetrics, Gynecology, and Reproductive Sciences, University of Miami Miller School of Medicine, Miami, USA; 2 Department of Internal Medicine, Hospital Corporation of America (HCA) Florida Kendall Hospital, Miami, USA; 3 Department of Biology, New York University (NYU) College of Arts and Science, New York, USA; 4 Department of Radiation Oncology, University of Miami Miller School of Medicine, Miami, USA; 5 Department of Pathology and Laboratory Medicine, University of Miami, Miami, USA

**Keywords:** placenta accreta, myometrium, oocyte, chorionic villi, increta, percreta

## Abstract

Advances in assisted reproductive technologies have enabled postmenopausal women to achieve pregnancy beyond their reproductive lifespan. Although rare, these pregnancies are challenging and require a multidisciplinary approach due to the higher prevalence of medical comorbidities in this population. The placenta accreta spectrum is characterized by an abnormal invasion of chorionic villi into the myometrium. Risk factors associated with the placenta accreta spectrum include prior uterine surgeries, advanced maternal age, multiparity, in vitro fertilization, and placenta previa. We present a case of a 59-year-old postmenopausal woman with chronic hypertension, stage II chronic kidney injury, and superimposed pre-eclampsia who underwent cesarean delivery complicated by suspected focal placenta accreta. Histopathological examination revealed significant deviations from normative placental architecture, emphasizing the invasion of the villi. Further, congested blood vessels and the presence of inflammatory cells, along with heightened collagen deposition, suggest an underlying pathological process affecting placental health. These findings underscore a perturbation of placental homeostasis, emphasizing the necessity for further investigation into the mechanisms contributing to placental pathology in postmenopausal pregnancies.

## Introduction

The past few decades have witnessed a sharp rise in maternal childbearing age [1]. Advances in the field of assisted reproductive technology (ART) have made it possible for women to conceive far beyond their natural reproductive life span. Menopause, which used to mark the end of the reproductive years, is no longer a limiting factor for conception. Although technologically feasible through ovum donation and in vitro fertilization (IVF), pregnancy after menopause carries several risks, given the increased prevalence of maternal comorbidities, including cardiovascular disease, hypertension, and diabetes [1]. The placenta accreta spectrum (PAS), previously known as morbidly adherent placenta, represents an abnormal trophoblast invasion of part or all of the placenta into the myometrium of the uterine wall [2]. Advanced maternal age during pregnancy is also associated with abnormal placentation, such as the placenta accreta spectrum. It is well known that disrupting the endometrial-myometrial interface can often lead to PAS [2]. By the same token, a postmenopausal milieu may lead to failure of normal decidualization and, ultimately, trophoblastic infiltration and abnormal placentation. However, no studies to date have been published on the association between PAS and the postmenopausal milieu. Given the comorbidities associated with postmenopausal pregnancy, the American Society for Reproductive Medicine (ASRM) has discouraged the use of donor embryos or oocytes in women older than 55 years of age [3]. Although postmenopausal pregnancies remain scarce, the number is expected to increase with time as more women begin to seek ovarian cryopreservation and consider utilizing their own preserved embryos or oocytes. We present a case of a 59-year-old postmenopausal female with chronic kidney disease and chronic hypertension who was conceived via oocyte donation and was found to have a focal placenta accreta.

## Case presentation

**Figure 1:**
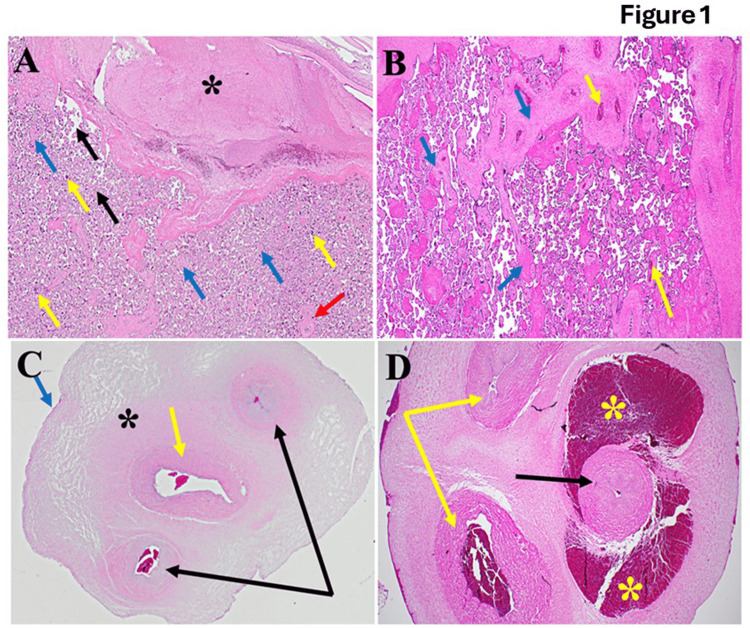
Architecture of placental tissues in normal and postmenopausal pregnancy women (A) Illustrating the typical architecture of placental tissues, the decidua is well-formed (black star). The intervillous space is clear, with few red blood cells (black arrow). Blue arrows point to the cytotrophoblast surrounded by syncytiotrophoblasts (yellow arrows). The red arrow points to villous blood vessels containing fetal blood. (B) shows diseased placental tissues; we observed significant disruption of the typical architecture of the decidua compared to Figure 1A (blue arrows). The yellow arrow indicates congested blood vessels. Additionally, scattered inflammatory cells and increased collagen deposition were noted. (C) The normal allantoic anatomy is illustrated by a single central artery (yellow arrow), paired veins (black arrows), and the black star, which indicates Wharton’s jelly. At the same time, the amniotic epithelium is marked by the blue arrow. On the other hand, this is evident in the fact that funisitis scatters diffusely (D). The artery (black arrow) is surrounded by severe congestion (yellow stars). Furthermore, inflammation of the veins' walls (phlebitis) and increased collagen deposition (yellow arrows) are noted.

A 59-year-old postmenopausal Haitian female, Gravida 4 Para 0 Aborta 3, presented to our institution for blood pressure control at 30 weeks of gestation. This was a pregnancy achieved via oocyte donation and was complicated by vanishing twin syndrome. Her last pregnancy resulted in an early miscarriage caused by dilation and curettage. The patient was known to have a history of Stage II chronic kidney disease and chronic hypertension maintained on methyldopa 250 mg three times daily. Upon presentation, the patient was noted to have systolic blood pressures ranging between 130 and 150 mmHg and diastolic blood pressures ranging between 60 and 80 mmHg. She was transitioned to nifedipine 30 mg daily. She presented again to us at 36 weeks of gestation with a persistent headache. She was found to have new-onset proteinuria and was diagnosed with chronic hypertension with superimposed pre-eclampsia with severe features. *An obstetrical ultrasound demonstrated a baby with an estimated fetal weight of 2045 grams in a transverse position and an unremarkable right lateral placenta, with the absence of features suggestive of placenta accreta (*Figure 1).* In light of her new diagnosis and fetal malpresentation, the decision was made to proceed with delivery via cesarean section.*

A Pfannenstiel incision was made, followed by dissection down to the fascia, which was then separated from the rectus abdominis both superiorly and inferiorly until reaching the pubic bone. Blunt entry was made into the peritoneum. Subsequently, a low transverse uterine incision was made, and a liveborn baby girl weighing 2.8 kilograms, with APGAR scores of 9/9/9, was delivered via breech extraction. Despite attempts at gentle traction on the umbilical cord, the placenta was found to be firmly adherent. Manual extraction was attempted, resulting in only partial removal of the placenta. The uterus was then exteriorized to improve visualization of the placenta and placental bed. Although the remaining portion of the placenta was visualized, attempts to remove it were unsuccessful, resulting in inward indentation and inversion of the uterus with each effort.

**Figure 2:**
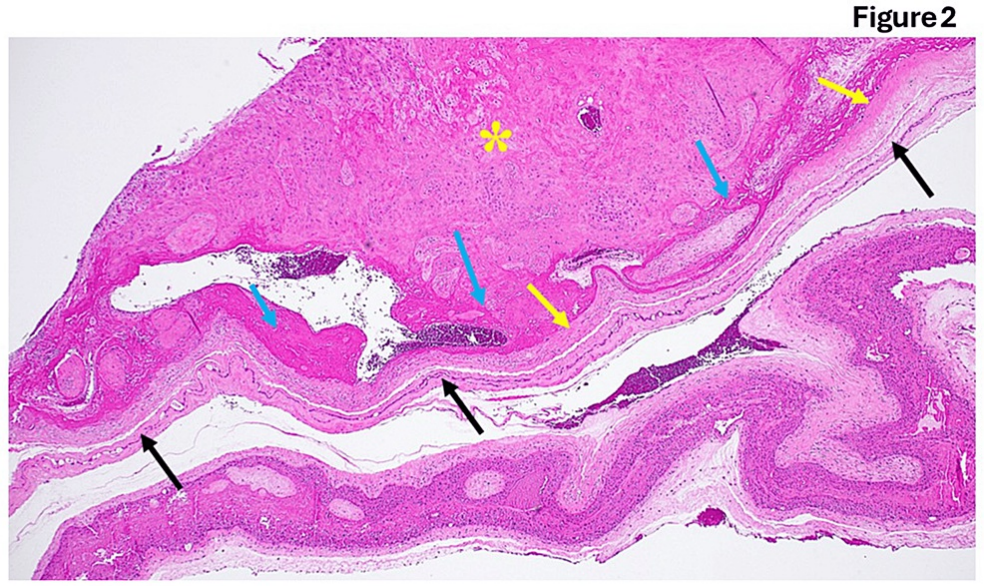
Fetal membranes from post-menopausal pregnancy There are different stages of amnion and chorion thickness (black and yellow arrows, respectively). Additionally, the trophoblasts appear malformed with significant collagen deposition. The decidual layer contains some intracellular edema (yellow star).

Intra-operatively, consultation with the gynecology oncology team was sought due to suspected focal placenta accreta. Tranexamic acid (1 gram) was administered, and a careful and progressive dissection of the placenta from the placental bed was performed, with the extracted tissue sent for pathological examination (Figures 1, 2). Following placental extraction, uterine atony was noted, and the myometrium was significantly thinned in the area where the placenta was noted to be deeply adherent. The patient received uterotonics, including intramuscular methergine (0.25mg) and hemabate (0.2mg). A right uterine artery ligation was performed. Total blood loss during the surgery amounted to 2036 ml. The patient's postpartum recovery was uneventful, and she was discharged on postoperative day four.

Histopathology

The normative architecture of placental tissues, as depicted in Figure 1A, showcases intricate histological features essential for supporting fetal development. Central to this structure is the decidua, a distinct maternal layer crucial for anchoring and nurturing the developing placenta. Within the expansive intervillous space, efficient hemotrophic exchange occurs, ensuring the transfer of nutrients, oxygen, and waste products between maternal and fetal circulations and sustaining fetal growth and metabolic demands. The coordinated interaction between cytotrophoblasts and syncytiotrophoblasts within the villous tree forms a dynamic syncytial barrier, regulating maternal-fetal exchanges and hormone synthesis essential for maintaining pregnancy homeostasis. Additionally, well-defined villous blood vessels play a pivotal role in establishing fetal circulation, completing the functional architecture of the placenta as a critical regulator of maternal-fetal physiology throughout gestation. Upon examination of diseased placental tissues in Figure 1B, notable deviations from the normative architecture are observed, particularly in the decidua, where significant disruptions are evident. Congested blood vessels, inflammatory cells, and heightened collagen deposition suggest an underlying pathological process affecting placental health. These findings underscore a perturbation of placental homeostasis, emphasizing the necessity for further investigation into the mechanisms contributing to placental pathology in diseased states.

**Figure 3:**
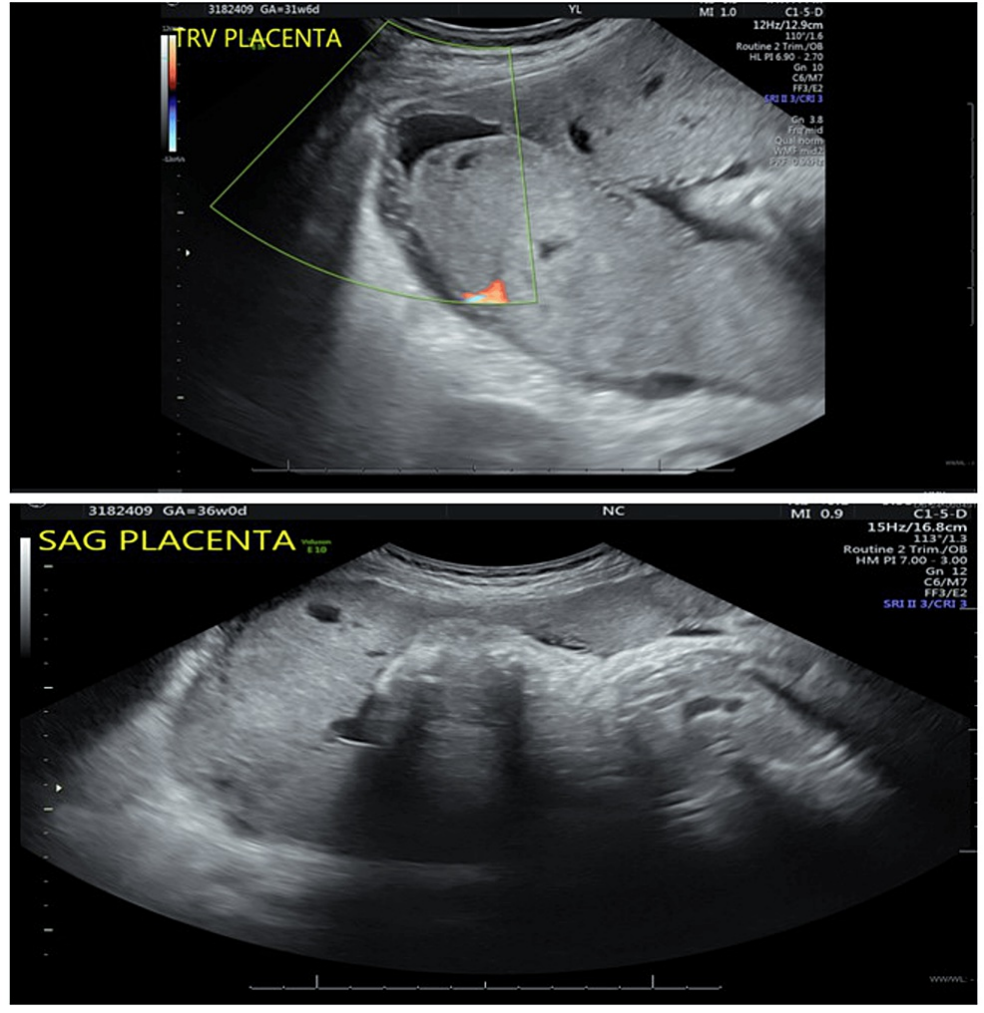
Placenta in both the transverse and sagittal plane The right lateral placenta shows no significant abnormalities.

Figure 1C illustrates the typical anatomy of the allantois, highlighting critical structures essential for fetal development. A central artery, paired veins, Wharton's jelly, and the amniotic epithelium form the structural framework facilitating nutrient and waste exchange. Wharton's jelly serves as a protective cushion around vessels, ensuring their integrity, while the amniotic epithelium maintains the amniotic fluid environment and provides mechanical protection to the fetus. In the presence of funisitis depicted in Figure 1D, pathological changes are evident within allantoic structures. Arterial congestion, phlebitis, and increased collagen deposition signify compromised blood flow, vascular inflammation, and tissue remodeling. These pathological features highlight the importance of investigating and managing conditions affecting the integrity and function of the allantois to mitigate potential adverse effects on fetal health. Figure 2 displays distinct variations in the thickness of the amnion and chorion layers, potentially reflecting developmental stages or pathological processes. Abnormalities in trophoblast morphology and intracellular edema within the decidual layer further indicate disrupted tissue architecture. Figure 3 depicts the ultrasound findings. These findings emphasize the dynamic nature of placental development and the significance of assessing histological features to discern normal from aberrant placental morphology.

## Discussion

ART has allowed a greater number of women to conceive in their later years, and with the use of donated oocytes, postmenopausal females can now even gestate and bear children [1]. However, this practice deviates from the usual practice, whereby ART is typically utilized to assist women who are infertile but otherwise premenopausal and relatively healthy. Although postmenopausal pregnancy via oocyte donation may be viewed as an extension of reproductive autonomy, physicians should evaluate and consider the risks the pregnancy poses to the patient and her fetus.

ASRM recommends that these women undergo a complete medical evaluation and thorough counseling regarding the risks of pregnancy [3]. Given the high prevalence of comorbidities with increasing maternal age, it is recommended that maternal-fetal specialists and internists evaluate these women during pregnancy to determine whether or not they can withstand the stressors of pregnancy. Our case not only highlights the high-risk nature of this pregnancy due to the patient’s complex comorbidities but also suggests the possibility that a postmenopausal uterus may constitute a hostile environment for the placenta. PAS includes a range of pathologic adherences of the placenta, including placenta accreta, increta, and percreta. A focally retained portion of the placenta that requires manual removal often represents a mild form of PAS. The diagnosis of PAS is confirmed by histology, which shows the absence of decidua and the presence of chorionic villi directly adjacent to myometrial fibers. Other histological findings include evidence of chronic basal inflammation, changes in maternal vascular malperfusion, and intramembranous and intervillous hemorrhage [4]. In this case, histopathology revealed disruption of the decidual plate, congested vessels, and intracellular edema within the decidual, consistent with PAS [4]. Several risk factors are associated with PAS, including a previous history of cesarean section, placenta previa, advanced maternal age, multiparity, conception via in vitro fertilization, and prior uterine surgeries or curettages. Our patient had several risk factors for PAS, including her conception by IVF, her age, and her history of dilation and curettage. It is difficult to determine which of the risk factors contributed most to her diagnosis of PAS. Several studies have investigated the effect of advanced maternal age (AMA) and very advanced maternal age (VAMA) on pregnancy outcomes, including placenta accreta; however, the pathologic mechanisms remain unknown [5-7]. In addition, studies have not examined the postmenopause period as a separate risk factor. Furthermore, postmenopausal pregnancies are conceived via ART, which is in and of itself associated with PAS; however, whether it is the postmenopausal milieu contributing to PAS or whether it is the altered cellular pathways during fertilization and early embryo culture occurring in vitro remains unknown [8]. Studies exploring the histopathology of these placentas remain scarce. More research is needed to determine whether the postmenopausal environment is an independent risk factor for PAS. Such research will aid in counseling postmenopausal women on pregnancy and the risk of placenta accreta at this age. A high index of suspicion for PAS should be maintained when evaluating a postmenopausal pregnancy. A multidisciplinary approach involving gynecology, oncology, and maternal-fetal medicine teams is recommended. In addition, we recommend a histological evaluation of the placenta after delivery for any postmenopausal woman, despite the absence of other risk factors.

## Conclusions

Placenta-accreta spectrum disorders pose substantial risks to maternal and fetal health, necessitating prompt recognition and effective management strategies. Although postmenopausal pregnancy has become feasible within the scope of ART, such pregnancies are considered “high-risk pregnancies” not only due to the complex existing comorbidities associated with advanced age but also due to the possibility of abnormal placentation. The presented case highlights the importance of a multidisciplinary approach, early detection of PAS, and postpartum evaluation of the placenta in these pregnancies. Thorough assessment and investigation of placenta accreta risk factors before embryo implantation should be performed in postmenopausal patients. Further research is needed to investigate the postmenopausal milieu as an independent risk factor for PAS to better counsel these patients.
